# Salvage surgery after chemotherapy with S-1 plus cisplatin for α-fetoprotein-producing gastric cancer with a portal vein tumor thrombus: a case report

**DOI:** 10.1186/1471-2482-15-5

**Published:** 2015-01-16

**Authors:** Shigetomi Nakao, Bunzo Nakata, Masashige Tendo, Kenji Kuroda, Takeshi Hori, Mayumi Inaba, Kosei Hirakawa, Tetsuro Ishikawa

**Affiliations:** Department of Surgery, Kashiwara Municipal Hospital, 1-7-9 Hozenji, Kashiwara City, Osaka, 582-0005 Japan; Department of Pathology, Osaka City University Graduate School of Medicine, 1-4-3 AsahimachiAbeno-ku, Osaka, 545-8585 Japan; Department of Surgical Oncology, Osaka City University Graduate School of Medicine, 1-4-3 AsahimachiAbeno-ku, Osaka, 545-8585 Japan

**Keywords:** Neoadjuvant chemotherapy, α-fetoprotein-producing gastric cancer, Portal vein tumor thrombus

## Abstract

**Background:**

Patient with α-Fetoprotein (AFP)-producing gastric cancer usually has a short survival time due to frequent hepatic and lymph node metastases. Gastric cancer with portal vein tumor thrombus (PVTT) is rare and has an extremely poor prognosis.

**Case presentation:**

A 63-year-old man was found to have a huge Type 3 gastric cancer with a PVTT and a highly elevated serum AFP level. Chemotherapy with S-1 plus cisplatin was given to this patient with unresectable gastric cancer for 4 months. The serum AFP level decreased from 6,160 ng/mL to 60.7 ng/mL with chemotherapy. Since the PVTT disappeared after the chemotherapy, the patient underwent total gastrectomy. Histological findings of the primary tumor after chemotherapy showed poorly differentiated adenocarcinoma without hepatoid cells and viable tumor cells remaining in less than 1/3 of the neoplastic area of mucosa and one lymph node. The cancerous cells were immunohistochemically stained by anti-AFP antibody. The patient has survived for 48 month without recurrence.

**Conclusions:**

AFP-producing gastric cancer with a PVTT has an extremely poor prognosis, but long-term survival was achieved for this dismal condition by salvage surgery after chemotherapy.

## Background

α-Fetoprotein-producing gastric cancer (AFP-GC) accounts for 1.6-4.3% of all gastric cancers and has biological aggressiveness, with frequent hepatic and lymph node metastases, resulting in a poor prognosis [1–4]. The mechanism of the aggressive behavior of AFP-GC has been under investigation. Recent molecular biological and genetic studies of AFP-CG have suggested that its malignancy is related to high vessel density [5], high expression of vascular epidermal growth factor (VEGF) [5], high expression of VEGF-C [6], frequent *p53* abnormalities [3], high expression of c-*Met* [7], absence of AT motif binding factor 1 [8], frequent loss of heterozygosity [9], and high fractional allelic loss in the tumor cells [9]. AFP-GC is pathologically divided into 2 types, hepatoid adenocarcinoma and nonhepatoid adenocarcinoma, including poorly/moderately/well-differentiated adenocarcinoma of common type gastric cancer and enteroblastic adenocarcinoma [10, 11]. Most tumors have both hepatoid and non-hepatoid components [10]. Although a portal vein tumor thrombus (PVTT) occurs frequently in hepatocellular carcinoma [12], it is rarely observed in gastric cancer [13]. The data of the 18^th^ follow-up survey of primary liver cancer by The Liver Cancer Study Group of Japan demonstrated that 26.1% of 5,368 patients with Hepatocellular carcinoma (HCC) had microscopic PVTT [14]. The annual report of the pathological autopsy cases in Japan showed that PVTT occurred in 1.2% of patients with gastric cancer [15]. It should be noted here that PVTT in liver cancer includes tumor thrombus in the intrahepatic portal vein, whereas PVTT in gastric cancer usually means thrombus limited to the main trunk or the first branch of the portal vein. Eom et al. reported that the median survival of patients with gastric cancer with PVTT was very short, at 5.4 months [16]. There have been few reports of salvage surgery after chemotherapy for AFP-GC in the English literature [17]. A case of AFP-GC with PVTT who has survived for 48 months without recurrence after salvage gastrectomy following chemotherapy with S-1 plus cisplatin is reported.

## Case presentation

**Figure 1:**
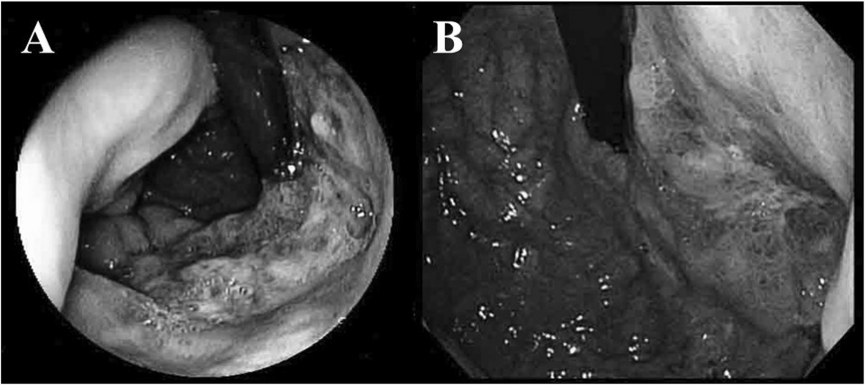
**Endoscopic imaging. (A)** Before chemotherapy with S-1 plus cisplatin **(B)** After 3 courses of chemotherapy.

**Figure 2:**
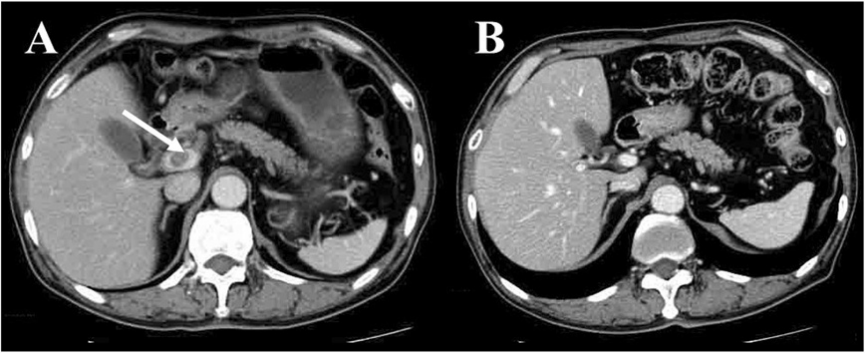
**Abdominal computed tomography imaging. (A)** Before chemotherapy with S-1 plus cisplatin **(B)** After 3 courses of chemotherapy.

**Figure 3:**
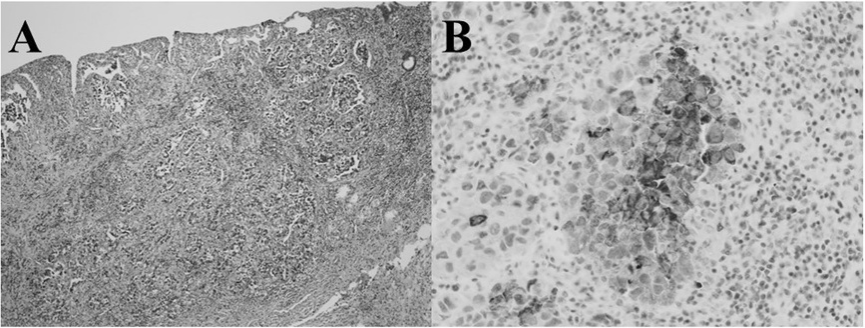
**Pathological findings. (A)** Hematoxylin-eosin staining (magnification X40). **(B)** Immunohistochemistry using anti-α-fetoprotein antibody.

**Figure 4:**
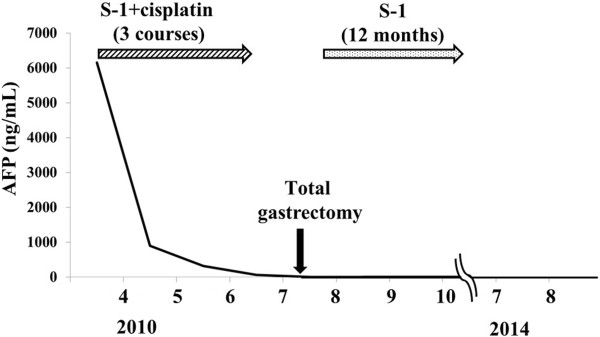
**The change in the serum α-fetoprotein value with salvage surgery following chemotherapy.**

A 63-year-old man was investigated for positive fecal occult blood in February 2010. Gastrointestinal endoscopy revealed a Type 3 gastric cancer located in the lesser curvature side and in middle and upper parts of the stomach. The oral side of the tumor was at 1cm anal side of esophagogastric junction (Figure 1A). The pathological diagnosis for biopsy specimens obtained by endoscopy was poorly differentiated adenocarcinoma of the stomach. Abdominal computed tomography (CT) showed a perigastric lymph node (30 mm × 23 mm) and a PVTT (16 mm × 13 mm). Endoscopic ultrasound was not performed because the PVTT was enhanced in the CT (Figure 2A). A PVTT is extremely rare in gastric cancer and has been reported to be associated with AFP-GC. Therefore, the serum α-Fetoprotein (AFP) level was measured and found to be very elevated (6,160 ng/mL). The patient had no liver diseases, including hepatitis and hepatocellular carcinoma. Gastric cancer with distant metastases, including a portal thrombus, has a very poor prognosis and is usually inoperable. This case was treated with combined chemotherapy of S-1 plus cisplatin, a standard regimen for unresectable gastric cancer in Japan [18]. After 3 courses of the regimen, composed of S1 (120 mg/day, orally, days 1-21) and cisplatin (60 mg/m^2^, intravenously, day 8) following by 14 days’ rest, the primary lesion was flattened (Figure 1B), the perigastric lymph node shrank to 13 mm × 10 mm, and the PVTT disappeared, allowing curative surgery to be performed (Figure 2B). The serum AFP level decreased to 60.7 ng/mL. Total gastrectomy and D2 lymphadenectomy was performed with the resection of distal esophagus to assure tumor-free margin. The macroscopic findings of the resected specimen included a slightly elevated tumor spreading from the cardia to the gastric body, with dimensions of 52 mm × 48 mm. The pathological diagnosis was poorly differentiated adenocarcinoma with invasion to the mucosa and one perigastric lymph node metastasis. No hepatoid cells were found in the resected specimens. The pathological staging of the current case was Stage IB, and the histological evaluation according to the criteria of tumor response of the Japanese Classification of Gastric Carcinoma, 3^rd^ English edition [19], after preoperative therapy was Grade 2 (viable tumor cells remained in less than 1/3 of the neoplastic area) (Figure 3A). Immunohistochemical staining showed AFP-GC (Figure 3B). S-1 was given for 12 months by the standard regimen of S-1 monotherapy (120 mg/day, orally, days 1-28, followed by 14 days’ rest). However, the amount was reduced to 80 mg/day after the second course due to Grade 3 diarrhea. The patient has been alive without recurrence for 48 months. The serum AFP level became 2.4 ng/mL after the operation and has continued within the normal limit (Figure 4).

## Conclusions

Inoue et al. reported that the 5-year survival rate of their 53-patient series of AFP-GC was 34%, although 53% of them had synchronous or metachronous hepatic metastases [4]. They suggested that the prognosis of AFP-GC was not as poor as previously believed, and that multimodality treatment may be useful to improve survival. Adachi et al. analyzed 270 cases of AFP-GC reported in the Japanese literature from 1982 to 2001, including one of their patients, and they reported that 5-year survival rates were 42% and 22%, and the median survival periods were 29 months and 14 months in patient with curative gastrectomy and in all patients, respectively [2]. Kochi et al. reported a significantly better response (70% vs. 31.9%, respectively), and a better conversion to surgery rate (40% vs. 12.8%) of stage IV AFP-CG by combination therapy with 5-fluorouracil, leucovorin, etoposide, and cisplatin was shown compared to Stage IV non-AFP-CG [17]. The disappearance of PVTT in the current AFP-GC by chemotherapy with S-1 plus cisplatin conferred operability on this case, resulting a long-term tumor-free survival after the curative operation. Based on the current case and the previous reports, salvage surgery for AFP-GC should be done to prolong survival when curative surgery could be performed after chemotherapy.

The current AFP-GC with PVTT had the elevated level of serum AFP and the poorly differentiated adenocarcinoma cells stained with anti-AFP antibody immunohistochemically. Although the incidence of PVTT in AFP-GC has not been elucidated, a few reports suggested a relatively high incidence of PVTT in AFP-GC. Araki et al. reported that, among four patients with gastric cancer and PVTT, three patients showed elevated serum AFP levels, and two patients were proven immunohistochemically to be producing AFP in the primary tumor [20]. Lee et al. found that 50% (4/8) of hepatoid adenocarcinomas of the stomach had PVTT in retrospective analyses of CT findings [21].

The prognosis of AFP-GC with PVTT has been thought to be miserable. However, Saitoh et al. [22] reported a case of AFP-GC with PVTT as a recurrent lesion after gastrectomy and following various kinds of chemotherapy for liver and lymph node metastases. The PVTT lesion showed partial response to irinotecan plus cisplatin as the 5^th^ line chemotherapy and S-1 monotherapy as the 6^th^ line chemotherapy, and the patient lived for more than 5 years after the initiation of systemic chemotherapy against recurrence involving the liver and lymph nodes. Contrary to the case reported by Saitoh et al, the PVTT occurred simultaneously with primary tumor and completely disappeared after chemotherapy in the current case. Both of the current and their cases demonstrated that chemotherapies for AFP-GC with PVTT were effective and contributed to the long survival. Yamaguchi et al. [23] reported 17 cases of AFP-GC with PVTT through a literature search from 1980 to 2002, including one of their own cases. They suggested that complete resection of AFP-GC with PVTT may lead to long-term survival based on their analysis of the literature data. The elimination of PVTT was an important factor for long term survival both in the current report and the case reported by Yamaguchi et al, although the removal methods of PVTT were quite different: chemotherapy and extirpation, respectively.

Recently, S-1 plus cisplatin for unresectable/recurrent gastric cancer has been recognized as a standard therapy in Japan [18]. A few phase II studies of neoadjuvant chemotherapy with S-1 plus cisplatin for advanced gastric cancer have been conducted [24, 25]. Following the promising results of these phase II studies, a phase III study (JCOG0501) of S-1 plus cisplatin as neoadjuvant chemotherapy for type 4 and large type 3 gastric cancer has been ongoing. The current case was expected to have a very poor prognosis at the time of diagnosis of AFP-GC with PVTT. However, chemotherapy with S-1 plus cisplatin was effective to decrease PVTT, and the primary lesion was curatively resected. This is the first report suggesting that salvage surgery following chemotherapy may contribute to curative resection of AFP-GC with PVTT.

## Consents

Written informed consent was obtained from the patient for publication of this Case report and any accompanying images. A copy of the written consent is available for review by the Editor of this journal.

## Abbreviations

AFP-GC: α-Fetoprotein-producing gastric cancer
VEGF: Vascular epidermal growth factor
PVTT: Portal vein tumor thrombus
HCC: Hepatocellular carcinoma
AFP: α-fetoprotein
CT: Computed tomography.
